# Self-reported work ability predicts health-related exit and absence from work, work participation, and death: longitudinal findings from a sample of German employees

**DOI:** 10.1007/s00420-020-01608-4

**Published:** 2020-11-21

**Authors:** Matthias Bethge, Katja Spanier, Stefanie Köhn, Anna Schlumbohm

**Affiliations:** 1 Institute for Social Medicine and Epidemiology, University of Lübeck, Ratzeburger Allee 160, 23562 Lübeck, Germany; 2 Institute of Medical Sociology and Rehabilitation Science, Charité, Universitätsmedizin Berlin, Berlin, Germany

**Keywords:** Needs assessment, Occupational health, Rehabilitation, Pensions, Cohort study

## Abstract

**Objective:**

The cohort study examined the performance of the Work Ability Index in predicting health-related exit and absence from work, work participation, and death among a sample of workers previously receiving sickness absence benefits.

**Methods:**

Workers aged 40–54 years who received sickness absence benefits in 2012 completed the Work Ability Index in 2013. Outcomes were extracted from administrative data records covering the period until the end of 2016.

**Results:**

Data for 2266 participants were included (mean age: 47.9 years; 54.4% women). Maximum follow-up was 43 months. In terms of work ability, 38.4% had good scores, 38.2% moderate scores, and 23.4% poor scores. Fully adjusted analyses showed an increased risk of a disability pension in workers with poor (HR = 12.98; 95% CI 5.81–28.99) and moderate Work Ability Index scores (HR = 3.17; 95% CI 1.36–7.38) compared to workers with good or excellent scores. The risk of a rehabilitation measure was also significantly increased for workers with poor and moderate scores. In addition, poor scores were prospectively associated with a longer duration of sickness absence and unemployment benefits, and fewer employment days and less income from regular employment. Those with poor Work Ability Index scores also had a significantly increased risk of premature death.

**Conclusions:**

The Work Ability Index is a potential tool to identify individuals with previous long-term sickness absence having an increased risk of health-related exit and absence from work and poor work participation outcomes.

**Electronic supplementary material:**

The online version of this article (10.1007/s00420-020-01608-4) contains supplementary material, which is available to authorized users.

## Introduction

The prevention of work disability and maintenance of work ability may require different actions, ranging from simple workplace adjustments to multi-component programmes. Particularly in cases with complex needs, coordinated care is essential to harmonise endeavours and services. This usually requires additional financial and staff resources. Risk-adjusted and stepped-care models are approaches that may achieve both efficient use of resources and access to coordinated care by establishing treatments of different levels of intensity. This model is well known from psychiatric care (Heddaeus et al. 2019) and has already been applied in occupational medicine and disability management (Aust et al. 2015; Poulsen et al. 2014; van Holland et al. 2012). In these models, the severity of symptoms or the prognosis determines the initial treatment choice. The patient’s course is closely monitored. If the patient does not respond to treatment, care will continue at the next level of intensity.

One important precondition of risk-adjusted and stepped-care models is an assessment that can stratify people according to their risk of permanent work disability so that it is possible to select a risk-adjusted intervention. In many countries, like the Netherlands, the Scandinavian countries, and Germany, the identification of workers in need of coordinated care is mainly directed by the duration of sickness absence (Mittag et al. 2018). In Germany, the employer has to initiate reintegration management if a worker is unable to work for a period of more than 6 weeks due to the same illness. Though sickness absence is a good predictor of permanent work disability, only a small proportion of sick-listed workers really needs sustained support. There is a clear need of an assessment that is suitable to identify workers who probably need lasting and intensive support. For this purpose, several tools have been proposed (Amler et al. 2018; Escorpizo et al. 2009; Leggett et al. 2016a, b). One of them is the Work Ability Index (WAI), a short, self-report screening tool, which is currently available in about 30 languages (Ilmarinen 2007, 2009). The WAI was developed by Ilmarinen and colleagues at the Finnish Institute of Occupational Health (Ilmarinen 2019). The questionnaire assesses the degree to which workers consider their state of health adequate to cope with their job demands. The continuous scores can be categorised into groups that reflect different levels of need of support.

There is emerging evidence from longitudinal studies that the WAI predicts work disability as measured by health-related early retirement (Alavinia et al. 2009a; Bethge et al. 2013, 2018; Jääskeläinen et al. 2016; Roelen et al. 2014; Tuomi et al. 1991) and long-term sickness absence (Ahlstrom et al. 2010; Alavinia et al. 2009b; Bethge et al. 2012, 2018; Kujala et al. 2006; Lundin et al. 2017; Reeuwijk et al. 2015; Schouten et al. 2015, 2016). These findings come mainly from Scandinavian countries and the Netherlands. For Germany, this kind of evidence is not yet well established. We, therefore, set out to validate these findings within the German social security scheme using administrative data on health-related exit and absence from work (disability pension, rehabilitation, sickness absence benefits), work participation (unemployment benefits, days in and income from employment), and death. For this purpose, we recruited a large cohort of workers with prior episodes of receipt of sickness absence benefits. Focusing on employees who had previously received sickness absence benefits for an absence of more than 6 weeks due to illness should ensure that we could test whether the WAI is suitable for risk stratification of persons who should be accompanied by occupational health services as part of the in Germany legally required reintegration management. In a previous paper using the baseline data of our study, we showed that self-reported work ability measured by the WAI was associated with a higher prevalence of occupational and behavioural health risks (Bethge et al. 2015). In another paper, we reported that the WAI predicted disability pensions, use of rehabilitation services, sickness absence, and unemployment benefits, as well as income from and days in regular employment after a follow-up of roughly one and a half years (Bethge et al. 2018). For the following analysis, we were able to consider an extended follow-up of nearly 4 years. Moreover, in addition to previous analyses, we could also extract data on mortality from administrative records. This is of importance, as there is only one previous study that investigated the association between the WAI and mortality (von Bonsdorff et al. 2011). In summary, we assumed that the WAI is associated with health-related exit and absence from work, work participation, and death.

## Methods

### Study design

The Third German Sociomedical Panel of Employees (GSPE-III) is a cohort study that was established to investigate the determinants of work ability, rehabilitation use, and receipt of disability pensions among employees who had previously received sickness absence benefits, a group that is particularly vulnerable to health-related early retirement (www.gspe3.de/en/) (Bethge et al. 2015). We used the STROBE checklist when preparing the manuscript to ensure transparent and complete reporting of our study design and findings (Vandenbroucke et al. 2007).

### Setting and participants

A sample of 10,000 people was drawn from the register of the Federal German Pension Insurance (GPI) (Bethge et al. 2015). This agency is part of the compulsory GPI scheme. In total, there are 16 agencies. The Federal GPI is the largest one and currently administers the pension contributions of around 23 million people (Deutsche Rentenversicherung Bund 2019). In the case of lasting work disability, the agencies have to pay a disability pension. Moreover, the pension insurance agencies can approve rehabilitation programmes for employees to improve and restore work ability and to avoid disability pensions. Sampling was restricted to those aged 40–54 years who had received sickness absence benefits in 2012. These benefits are usually paid in the case of sick leave episodes lasting more than 6 weeks. Those who had previously made pension requests were excluded, as were individuals who had requested or used rehabilitation services during the last 4 years. Men and women were sampled independently. The baseline questionnaires were sent in May 2013, and in the case of non-return, they were followed by one reminder 6 weeks later. If responders gave their approval, survey data were linked to administrative data records. Follow-up data from administrative records covering the years 2013–2016 were provided by the Federal GPI at the end of 2015 and 2017. These data came from the rehabilitation statistic data set that for a given year is available at the end of the following year. The study protocol was approved by the ethics committee of the Hannover Medical School (1730–2013) and the data protection commissioner of the Federal GPI. The GSPE-III was registered in the German Clinical Trials Register (DRKS00004824).

### Outcomes

All outcome data were extracted from the administrative records of the Federal GPI. Administrative data were used to avoid recall bias and response bias due to selective sample attrition. Data on disability pensions, rehabilitation measures, and death covered the period from study entry in 2013 until the end of 2016. In addition to these events, we also considered the time at risk for these events in our analyses. Data on welfare benefits due to sickness absence and unemployment and data on employment were added for the years 2015 and 2016.

### Work Ability Index

Work ability was assessed using the German version of the WAI questionnaire (Ilmarinen 2007). This self-report measure comprises seven scores, which are derived from 11 items: current work ability compared with lifetime best; work ability in relation to the physical and mental demands of the job (two items and an additional item to weigh physical and mental demands); number of current diseases diagnosed by a physician; estimated work impairment due to disease; sick leave during the past year; own prognosis of work ability 2 years from now; and mental resources (three items). The English version of the German questionnaire used in our study can be found elsewhere (Bethge et al. 2015). The total WAI score ranges from 7 to 49 points. Higher scores indicate better work ability. Levels of work ability can be categorised as poor (7–27 points), moderate (28–36 points), good (37–43 points), and excellent (44–49 points). We merged the upper categories to form one category of good scores due to the rather small number of outcome events in these categories. In line with Alavinia et al. (2009a) we distinguished among three levels of work ability when predicting our outcomes.

### Covariates

Self-reported data and administrative data on demographics, work and work environment, health behaviour, and welfare benefits were considered as covariates. Age and sex were derived from administrative records. Self-reported demographic data comprised educational level (low, moderate, high) and partnership (partnered vs. single). Our categorisation of the educational level was based on the degrees that are possible in Germany. The German system distinguishes two lower secondary degrees which were categorised as low or moderate, and an upper secondary degree that enables access to tertiary education. Self-reported data on work and work environment covered volume of employment (full-time or part-time), job demands (mental work, physical work, an equal amount of mental and physical work) (Ilmarinen 2007), job position (blue collar vs. white collar) (Bethge et al. 2015), and size of enterprise (< 50 employees, 50–249 employees, ≥ 250 employees) (European Commission 2015). Self-reported data on health behaviour included smoking (never smoker, current smoker, former smoker), sports (≥ 2 h per week vs. < 2 h per week), and obesity (body mass index ≥ 30 vs. body mass index < 30). The body mass index was calculated from self-reported height and weight. Welfare benefits due to sickness absence and unemployment, as well as days in employment and income from employment, were extracted from administrative records and covered the years 2011 and 2012.

### Study size

The GSPE-III is a cohort study to explore a range of research questions related to the use of rehabilitation services and disability pensions. To enable us to follow up on rehabilitation use and disability pension for at least 3000 participants in the baseline survey, we drew a sample of 10,000 people. Assumptions regarding the response rate and the rate of consent to link survey and administrative data were derived from previous studies (Bethge and Radoschewski 2012; Bethge et al. 2012).

### Statistical analysis

Descriptive statistics were used to characterise the full sample and samples stratified on the basis of levels of work ability. To describe the risk of disability pensions, rehabilitation, and death, we calculated absolute risks and rates per 1000 person-years. Time at risk was computed from the date of receipt of the questionnaire. Observations were censored at the end of 2016, and in case of disability pensions and rehabilitation also at the date of death. Kaplan–Meier curves were examined to compare the cumulative probability of disability pensions, rehabilitation, and death according to the level of work ability during the follow-up. Proportional hazard models were fitted to determine the supplementary prognostic benefit of the WAI when considering relevant covariates, and the hazard ratio (HR) and corresponding 95% CI were estimated. We first calculated crude associations and then subsequently added age and sex, administrative data on welfare benefits and work participation in 2011 and 2012, and self-reported data from the baseline survey in 2013. To determine the associations between the WAI levels and sickness absence benefits and work participation outcomes in 2015 and 2016, linear regression models were fitted. All the covariates mentioned above were included in the final, fully adjusted models.

Missing self-reported baseline data were imputed using chained equations (Royston and White 2011). Parameters without missing values (age, sex, job demands, and administrative data) were included as covariates in the imputation model. We created 20 independent data sets with complete values. Parameter estimates of the proportional hazard and the linear regression models were combined in accordance with Rubin’s rules (Little and Rubin 2002).

The statistical test results were regarded as significant if the two-sided *p* value of a test was less than 0.05. All calculations were performed in Stata SE 15.

## Results

### Recruitment and participants

Of the 10,000 questionnaires sent, 103 could not be delivered. Completed questionnaires were returned by 3294 (33.3%) individuals. The responders were marginally older than non-responders (47.9 vs. 47.2 years) and slightly more likely to be female (53.6% vs. 48.4%). We excluded 305 participants due to their unemployment by the time of the initial survey, six participants due to missing information concerning their employment status, and 170 participants due to missing data for self-reported work ability. Of the remaining 2813 people, 2342 (83.3%) participants agreed to the linking of questionnaire data and administrative data records, whereas 471 participants refused. Sixty-seven persons were excluded because they changed their pension agency, and data about them was no longer available from the Federal GPI. Nine subjects were excluded because they applied for a disability pension benefit before the baseline questionnaire was registered by the research team. In total, 2266 participants were eligible for analysis related to disability pensions, rehabilitation, and death. In the case of the data on sickness absence and unemployment benefits, and employment data, 146 additional subjects had to be excluded due to incomplete administrative data records. Online Resource 1 shows the flow of participants.

Table 1 presents selected baseline characteristics and outcomes of the total sample and stratified for self-reported work ability. The mean age was 47.9 years, and 54.4% were women. In terms of work ability, 38.4% had good scores, 38.2% moderate scores, and 23.4% poor scores. The complete table of all baseline characteristics is available as Online Resource 2.

**Table 1:** Selected baseline characteristics and outcomes: all respondents and stratified based on levels of work ability

	Total (*n* = 2266)			Poor (*n* = 532)			Moderate (*n* = 865)			Good/excellent (*n* = 869)		
	*n*	%	Mean (SD)	*n*	%	Mean (SD)	*n*	%	Mean (SD)	*n*	%	Mean (SD)
Baseline												
Sex												
Male	1033	45.6		241	45.3		387	44.7		405	46.6	
Female	1233	54.4		291	54.7		478	55.3		464	53.4	
Age	2266		47.9 (4.1)	532		48.1 (4.2)	865		48.0 (4.1)	869		47.6 (4.1)
40–44 years	548	24.2		127	23.9		198	22.9		223	25.7	
45–49 years	826	36.5		176	33.1		315	36.4		335	38.6	
50–54 years	892	39.4		229	43.0		352	40.7		311	35.8	
Employment												
Full-time	698	31.1		183	34.8		270	31.5		245	28.5	
Part-time	1546	68.9		343	65.2		588	68.5		615	71.5	
Missing	22			6			7			9		
Outcomes												
Disability pension												
Yes	94	4.1		62	11.7		25	2.9		7	0.8	
No	2172	95.9		470	88.3		870	97.1		862	99.2	
Rehabilitation												
Yes	471	20.8		195	36.7		181	20.9		95	10.9	
No	1795	79.2		337	63.3		684	79.1		774	89.1	
Death												
Yes	29	1.3		14	2.6		12	1.4		3	0.3	
No	2237	98.7		518	97.4		853	98.6		866	99.7	
Days with sickness absence benefits in 2015 and 2016	2120		32.9 (88.6)			57.1 (114.7)			33.6 (90.2)			18.1 (62.4)
Days with unemployment benefits in 2015 and 2016	2120		18.0 (64.0)			43.0 (97.4)			12.1 (53.6)			9.1 (41.1)
Days in employment in 2015 and 2016	2120		67,246 (40,586)			52,440 (40,502)			69,536 (39,868)			73,664 (39,210)
Income from employment in euros in 2015 and 2016	2120		620.4 (225.5)			512.1 (293.5)			632.8 (210.5)			671.5 (164.7)

*SD* standard deviation

### Disability pensions, rehabilitation, and death

**Fig. 1:**
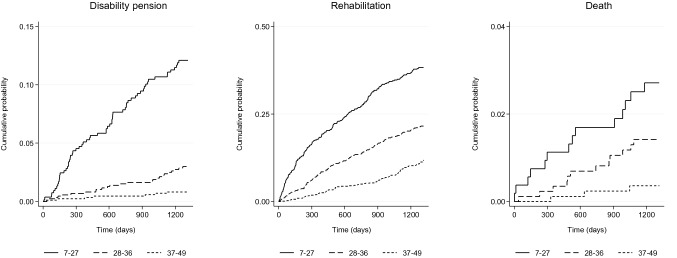
Cumulative probability of disability pension, rehabilitation, and death based on levels of self-reported work ability during the follow-up. *n* = 2266

During the maximum follow-up of 43 months (3.6 years), 94 participants (4.1%) were approved a disability pension, 471 (20.8%) were approved a rehabilitation measure, and 29 (1.3%) died. The absolute risks of receiving a disability pension were 11.7, 2.9, and 0.8% for those with poor, moderate, and good work ability, respectively. The risks of a rehabilitation event were 36.7, 20.9, and 10.9%; the risks of death were 2.6, 1.4, and 0.3%. Table 2 reports the incidence rates per 1000 person-years, and Fig. 1 shows the cumulative probability of approved disability pensions, rehabilitation, and death.

**Table 2:** Incidence rate per 1000 person-years of disability pensions, rehabilitation, and death: all respondents and stratified based on levels of self-reported work ability

	Total (*n* = 2266)		Poor (*n* = 532)		Moderate (*n* = 865)		Good/excellent (*n* = 869)	
	IR	95% CI	IR	95% CI	IR	95% CI	IR	95% CI
Disability pension	12.35	10.09; 15.11	36.75	28.65; 47.14	8.49	5.74; 12.57	2.35	1.12; 4.92
Rehabilitation	68.85	62.90; 75.36	143.74	124.92; 165.40	68.84	59.51; 79.64	33.27	27.21; 40.68
Death	3.74	2.60; 5.38	7.86	4.65; 13.27	4.03	2.29; 7.09	1.00	0.32; 3.11

*n* = 2266; *IR* incidence rate per 1000 person-years; *CI* confidence interval

The adjusted risk estimates from the proportional hazard models are presented in Table 3. The results show that the associations between baseline work ability and disability pensions, rehabilitation, and death were only slightly reduced when adjusting for age and sex, baseline data from the administrative records, and self-reported data. In the final, fully adjusted model, poor work ability was associated with an approximately 13 times greater risk (HR = 12.98; 95% CI 5.81–28.99) of a disability pension during follow-up than good work ability. Moderate work ability was associated with a risk that was about 3 times as great (HR = 3.17; 95% CI 1.36–7.38). The risk of a rehabilitation event during follow-up was 4 times greater for workers with poor work ability (HR = 3.90; 95% CI 3.02–5.05) and twice for workers with moderate work ability (HR = 1.91; 95% CI 1.48–2.46). Moreover, poor work ability was associated with a risk of death 6 times greater (HR = 6.19; 95% CI 1.70–22.53), while the risk for persons with moderate work ability did not significantly increase.

**Table 3:** Association between self-reported work ability and disability pension, rehabilitation, and death in the period 2013–2016: hazard ratios and 95% confidence intervals

	Disability pension			Rehabilitation			Death		
	HR	95% CI	*p*	HR	95% CI	*p*	HR	95% CI	*p*
Crude									
Poor	15.57	7.13; 34.02	< 0.001	7.80	2.24; 27.15	0.001	4.25	3.33; 5.44	< 0.001
Moderate	3.61	1.56; 8.36	0.003	4.02	1.13; 14.24	0.031	2.06	1.61; 2.64	< 0.001
Adjusted for age and sex									
Poor	15.12	6.92; 33.06	< 0.001	7.65	2.20; 26.65	0.001	4.17	3.26; 5.33	< 0.001
Moderate	3.49	1.51; 8.07	0.004	3.98	1.12; 14.11	0.033	2.01	1.57; 2.57	< 0.001
Additionally adjusted for administrative data									
Poor	13.09	5.92; 28.94	< 0.001	6.26	1.76; 22.35	0.005	4.04	3.14; 5.20	< 0.001
Moderate	3.29	1.42; 7.63	0.005	3.67	1.03; 13.10	0.045	1.98	1.54; 2.54	< 0.001
Additionally adjusted for self-reported data									
Poor	12.98	5.81; 28.99	< 0.001	6.19	1.70; 22.53	0.006	3.90	3.02; 5.05	< 0.001
Moderate	3.17	1.36; 7.38	0.007	3.55	0.99; 12.78	0.053	1.91	1.48; 2.46	< 0.001

*n* = 2266; *HR* hazard ratio, *CI* confidence interval; estimates from the final, fully adjusted were calculated using imputed data from 20 imputed data sets

### Sickness absence benefits and work participation

Table 4 shows the adjusted estimates of the prognostic relevance of the work ability categories for sickness absence benefits and work participation outcomes in 2015 and 2016. Compared to participants with good work ability, those with poor work ability had 32 additional days of sickness absence benefits and 32 additional days of unemployment benefits. Moreover, days in employment were reduced by 151 days among participants with poor work ability; the 2-year income was reduced by 16,566 euros. Participants with moderate work ability had 11 additional days of sickness absence benefits when compared to those with good work ability, while days in employment were decreased by 39 days and the 2-year income was 3548 euros less.

**Table 4:** Association between self-reported work ability and sickness absence benefits and work participation outcomes in 2015 and 2016: adjusted mean differences and 95% confidence intervals

	Poor vs. good work ability		Moderate vs. good work ability	
	*b*	95% CI	*b*	95% CI
Days with sickness absence benefits in 2015 and 2016	32.3	22.0; 42.6	11.3	2.7; 19.9
Days with unemployment benefits in 2015 and 2016	31.8	24.4; 39.1	2.5	− 3.6; 8.7
Days in employment in 2015 and 2016	− 151.0	− 175.1; − 126.8	− 39.4	− 59.7; − 19.2
Income from employment in 2015 and 2016	− 16,566	− 19,465; − 13,666	− 3548	− 5974; − 1123

*n* = 2120; *b* unstandardised estimate, *CI* confidence interval; estimates are adjusted for age, sex, education, partnership, employment, job demands, job position, size of enterprise, smoking, body mass index, sports activity, days with sickness absence benefits (2011 and 2012), days with unemployment benefits (2011 and 2012), days in employment (2011 and 2012), and income from employment in euros (2011 and 2012). Estimates of the final, fully adjusted model were calculated using imputed data from 20 imputed data sets

## Discussion

Participants with poor and moderate WAI scores had an elevated risk of permanent work disability and premature death, longer periods of sickness absence benefits, and shorter periods of employment and less income from employment even after adjusting for a range of other variables. Poor WAI scores were also associated with an increased likelihood of using rehabilitation services and longer periods of receiving unemployment benefits.

Our findings are consistent with the emerging evidence from large high-quality cohort studies, which started in Scandinavian countries and the Netherlands in the 1980s to clarify if the WAI is able to identify workers with an increased risk of permanent work disability. Jääskeläinen et al. (2016) recently reported that the WAI predicted disability pensions in a cohort of 5251 Finnish municipal employees. The adjusted HRs were quite similar to our estimates. Roelen et al. (2014) used the continuous WAI score to predict disability pensions in 9350 Dutch workers and found that the continuous score discriminated well between workers with and without future disability pensions. Schouten et al. in two publications and Reeuwijk et al. showed reasonable performance of the WAI in predicting sickness absence spells of different durations drawing on large Dutch samples of more than 1000 people (Reeuwijk et al. 2015; Schouten et al. 2015, 2016). Additionally, there is strong evidence from two large Swedish cohort studies that the WAI and its single items are associated with an increased risk of a disability pension (Lundin et al. 2016, 2017). Moreover, poor work ability as measured with the first item of the WAI may impact beyond retirement. Bonsdorff et al. (2011) showed with 28-year follow-up data from the Finnish Longitudinal Study of Municipal Employees that poor work ability—as in our study—was associated with mortality, and that poor work ability nearly doubled the risk of some form of disability when performing activities of daily living as senior citizens.

Efficient use of resources when managing occupational health services can be supported by providing a risk-adjusted selection of pathways instead of relying on one-size-fits-all measures. This assures that unnecessary efforts in individuals with a low risk are abandoned while individuals with a high risk receive the intense support that they need. Our findings indicate that the WAI is a prognostic relevant tool to identify who is at risk and who needs intense and coordinated care.

A critical appraisal of the findings of this study has to consider the following limitations. First, the response rate of only approximately one-third reflects a risk of bias, although a comparison of responders and non-responders showed only minor differences related to age and sex. Second, the analyses were restricted to participants who approved the linking of questionnaire data and administrative data records. This further limited the sample, as only 83.3% agreed to this data linkage. Third, the workers in our sample were predominantly employed in the public sector. This was the result of sampling participants from the register of the Federal GPI. Workers who are part of this pension scheme are characterised by higher educational levels, higher vocational qualifications, and higher income, and they are less frequently exposed to high physical demands in the workplace compared to the general population. Fourth, we used the categorized WAI total score in our analyses, only. We showed that the categorized WAI total score is a robust prognostic measure considering a range of work participation outcomes. However, depending on the outcome, the single dimensions of the WAI may have a similar prognostic value as the total score. Additional analyses are needed to clarify, for which outcomes single dimensions of the WAI may be sufficient to predict work participation outcomes, and to what amount the prognostic value is driven by the seven single dimensions (Reeuwijk et al. 2015). Finally, several models to predict work disability were published in recent years. While these models were useful to identify risk factors, they were mostly not good enough to validly determine who is likely to leave the workforce. Burdorf (2019) suggested that occupational health care professional should focus their efforts on reducing well-established and widespread risk factors, rather than developing even more sophisticated predictive models to identify high-risk individuals as accurately as possible.

These limitations are balanced by several strengths. First, the sample was drawn from the register of the country’s largest pension body, the Federal GPI, and was restricted to those who had previously received sickness benefits (i.e., a group particularly at risk of early retirement and permanent work disability, and, therefore, in need of effective occupational health care). The distribution of the WAI categories, therefore, differed clearly from other studies using the WAI to predict work participation outcomes, and people with poor and moderate WAI scores were overrepresented (Jääskeläinen et al. 2016; Reeuwijk et al. 2015; Roelen et al. 2014). Second, the linking of the questionnaire data and administrative data records allowed follow-up without sample attrition. Third, the use of administrative data allowed complete, valid, and reliable assessment of our study outcomes. Thus, recall bias and misclassification were avoided. Fourth, our extended follow-up was nearly 4 years, and the median time to claim a disability pension in case of an event was roughly 19 months. We are confident that this time span would enable to offer complex multi-component programmes to prevent disability pensions.

In conclusion, the WAI groups workers into categories with different risks of permanent work disability and may be used to organise risk-adjusted and stepped-care models in occupational health care settings.

## Electronic supplementary material

Below is the link to the electronic supplementary material.

Flow of participants (PDF 10 KB)

Baseline characteristics: all respondents and stratified based on levels of work ability (PDF 139 KB)

## Data Availability

All data relevant to the study are included in the article or uploaded as supplementary information.

## Code availability

The code generated to analyse the data is available from the corresponding author on reasonable request.
