# Supplementary figures and images for: Self-reported work ability predicts health-related exit and absence from work, work participation, and death: longitudinal findings from a sample of German employees
Source: Int Arch Occup Environ Health. 2020 Nov 21;94(4):591–9. doi: 10.1007/s00420-020-01608-4 (PMC8068707; doi:10.1007/s00420-020-01608-4)

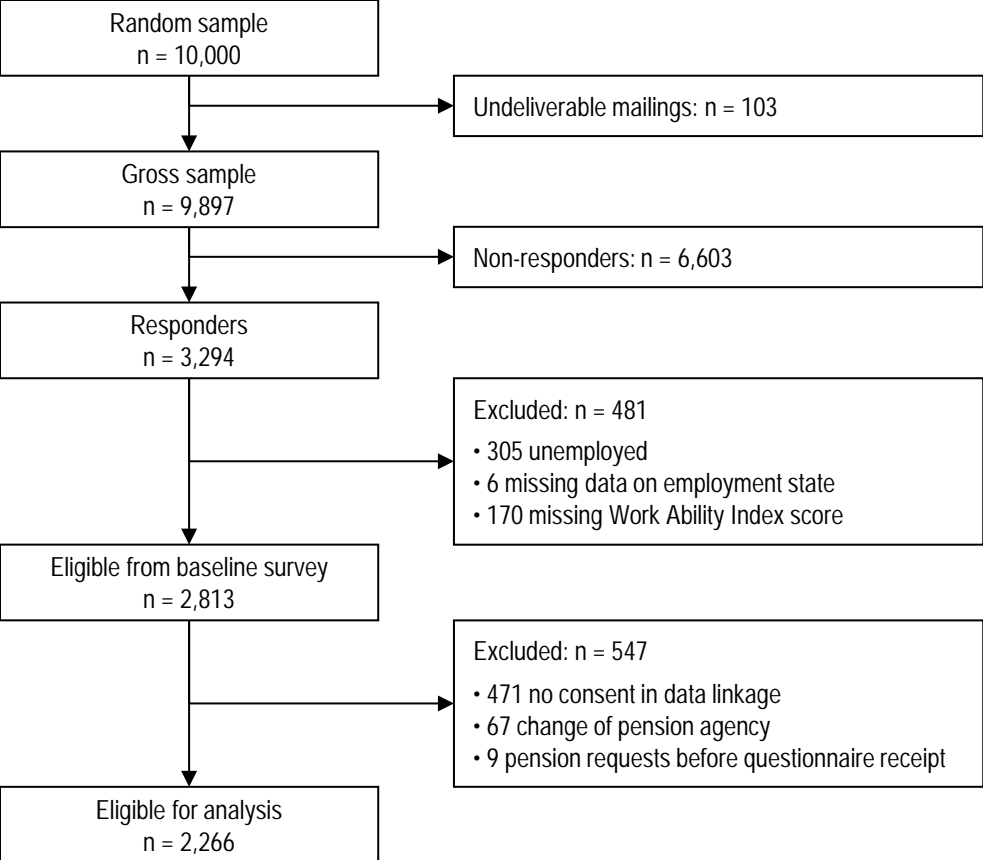

Supplement: Supplementary file 1 — Flow of participants (PDF 10 KB) [file 420_2020_1608_MOESM1_ESM.pdf]
