# Supplementary material for: Self-reported work ability predicts health-related exit and absence from work, work participation, and death: longitudinal findings from a sample of German employees
Source: Int Arch Occup Environ Health. 2020 Nov 21;94(4):591–9. doi: 10.1007/s00420-020-01608-4 (PMC8068707; doi:10.1007/s00420-020-01608-4)
Supplement: Supplementary file 2 — Baseline characteristics: all respondents and stratified based on levels of work ability (PDF 139 KB) [file 420_2020_1608_MOESM2_ESM.pdf]

Online Resource 2 Baseline characteristics: all respondents and stratified based on levels of work ability

	Total (n = 2,266)			Poor (n = 532)			Moderate (n = 865)			Good/excellent (n = 869)		
	n	%	Mean (SD)	n	%	Mean (SD)	n	%	Mean (SD)	n	%	Mean (SD)
Sex												
Male	1,033	45.6		241	45.3		387	44.7		405	46.6	
Female	1,233	54.4		291	54.7		478	55.3		464	53.4	
Age	2,266		47.9 (4.1)	532		48.1 (4.2)	865		48.0 (4.1)	869		47.6 (4.1)
40–44 years	548	24.2		127	23.9		198	22.9		223	25.7	
45–49 years	826	36.5		176	33.1		315	36.4		335	38.6	
50–54 years	892	39.4		229	43.0		352	40.7		311	35.8	
Education												
Low	453	20.1		155	29.3		166	19.3		132	15.2	
Medium	1,342	59.4		287	54.3		526	61.0		529	60.9	

High	464	20.5		87	16.4		170	19.7		207	23.8	
Missing	7			3			3			1		
Partnership												
Partnered	1,748	78.8		400	77.1		651	77.0		697	81.5	
Single	471	21.2		119	22.9		194	23.0		158	18.5	
Missing	47			13			20			14		
Employment												
Full-time	698	31.1		183	34.8		270	31.5		245	28.5	
Part-time	1,546	68.9		343	65.2		588	68.5		615	71.5	
Missing	22			6			7			9		
Job demands												
Mental	1,097	48.4		239	44.9		416	48.1		442	50.9	
Mental and physical	966	42.6		230	43.2		371	42.9		365	42.0	
Physical	203	9.0		63	11.8		78	9.0		62	7.1	

Job position												
Blue collar	204	9.1		54	10.2		82	9.5		68	7.9	
White collar	2,047	90.9		473	89.8		777	90.5		797	92.1	
Missing	15			5			6			4		
Size of enterprise												
< 50 employees	698	31.2		173	32.8		244	28.6		281	32.9	
50–249 employees	496	22.2		120	22.8		185	21.7		191	22.4	
≥ 250 employees	1,041	46.6		234	44.4		425	49.8		382	44.7	
Missing	31			5			11			15		
Smoking												
Never smoker	896	39.6		188	35.4		345	39.9		363	42.0	
Current smoker	649	28.7		177	33.3		239	27.7		233	26.9	
Former smoker	715	31.6		166	31.3		280	32.4		269	31.1	
Missing	6			1			1			4		

Body mass index												
< 30	1,746	77.5		380	71.7		645	75.1		721	83.5	
≥ 30	506	22.5		150	28.3		214	24.9		142	16.5	
Missing	14			2			6			6		
Sports activity												
< 2 hours	848	37.5		151	28.6		295	34.1		402	46.4	
≥ 2 hours	1,411	62.5		377	71.4		569	65.9		465	53.6	
Missing	7			4			1			2		
Days with sickness absence benefits in 2011 and 2012	2,266		84.3 (83.1)	532		112.9 (99.4)	865		87.0 (84.1)	869		64.1 (63.4)
Days with unemployment benefits in 2011 and	2,266		8.9 (39.1)	532		4.7 (30.0)	865		7.9 (34.9)	869		12.4 (46.9)

2012												
Days in employment in 2011 and 2012	2,266		631.5 (139.2)	532		610.4 (144.7)	865		632.3 (135.4)	869		643.5 (138.1)
Income from employment in euros in 2011 and 2012	2,266		60,669 (31,581)	532		56,293 (28,586)	865		61,184 (31,010)	869		62,835 (33,596)

SD = standard deviation
